# Treatment plan comparison of volumetric‐modulated arc therapy to intensity‐modulated radiotherapy in lung stereotactic body radiotherapy using either 6‐ or 10‐MV photon energies

**DOI:** 10.1002/acm2.13714

**Published:** 2022-07-09

**Authors:** Zhigong Wei, Xingchen Peng, Ling He, Jingjing Wang, Zheran Liu, Jianghong Xiao

**Affiliations:** ^1^ Department of Biotherapy, Cancer Center West China Hospital Sichuan University Chengdu Sichuan China; ^2^ Department of Radiation Oncology, Cancer Center West China Hospital Sichuan University Chengdu Sichuan China

**Keywords:** intensity‐modulated radiotherapy (IMRT), lung cancer, radiotherapy, stereotactic body radiotherapy (SBRT), volumetric‐modulated arc therapy (VMAT)

## Abstract

**Purpose:**

The aim of this study was to dosimetrically compare volumetric‐modulated arc therapy (VMAT) with intensity‐modulated radiotherapy (IMRT) techniques using either 6‐ or 10‐MV photon beam energies in lung stereotactic body radiation therapy (SBRT) plans.

**Methods:**

Thirty patients with primary or metastatic lung tumors eligible for SBRT were randomly selected. VMAT and IMRT treatment plans using either 6‐ or 10‐MV photon energies were generated through automatic SBRT planning software in the RayStation treatment planning system.

**Results:**

For planning target volume, there was no difference in *D*_95%_ for all plans, whereas *D*_2%_ and *D*_50%_ were significantly increased by 5.22%–5.98% and 2.47%–2.59%, respectively, using VMAT_6/10‐MV_ plans compared to IMRT_6/10‐MV_ plans. When comparing the *D*_max_ of organs at risk (OARs), VMAT_6/10‐MV_ was 18.32%–47.95% lower than IMRT_6/10‐MV_ for almost all OARs. VMAT_6/10‐MV_ obviously decreased *D*_mean_, *V*_5Gy_, *V*_10Gy_, and *V*_20Gy_ of whole lung by 9.68%–20.92% than IMRT_6/10‐MV_. Similar results were found when comparing VMAT_6‐MV_ with IMRT_10‐MV_ or VMAT_10‐MV_ with IMRT_6‐MV_. The differences in the *D*_2%_, heterogeneity index, and conformity index between 6‐ and 10‐MV plans are not statistically significant. Plans using 6‐MV performed 4.68%–8.91% lower levels of *D*_max_ of spinal cord, esophagus, great vessels, and trachea and proximal bronchial tree than those using 10‐MV plans. Similarly, *D*_mean_, *V*_5Gy_, *V*_10Gy_, and *V*_20Gy_ of whole lung were also reduced by 2.79%–5.25% using 6‐MV. For dose fall‐off analysis, the *D*_2cm_ and *R*_50%_ of VMAT_6/10‐MV_ were lower than those of IMRT_6/10‐MV_. Dose fall‐off curve based on 10 rings was steeper for VMAT plans than IMRT plans regardless of the energy used.

**Conclusions:**

For lung SBRT plans, VMAT‐based plans significantly reduced OARs dose and steepened dose fall‐off curves compared to IMRT‐based plans. A 6‐MV energy level was a better choice than 10‐MV for lung SBRT. In addition, the dose differences between different techniques were more obvious than those between different energy levels.

## 1 INTRODUCTION

Stereotactic body radiation therapy (SBRT), as an external and noninvasive cancer treatment, uses accurate radiation beams to deliver a high dose of radiation in one to five fractions with 8–30 Gray (Gy) per fraction to treat tumors.^1^, ^2^ SBRT is a standard treatment option for medically inoperable, early‐stage non‐small cell lung cancer (NSCLC), with a high rate of local control and low treatment‐related toxicity.^3^, ^4^, ^5^ SBRT could also be a promising and comparable radical treatment for operable cases and pulmonary metastases.^5^, ^6^, ^7^, ^8^ However, uncertainty still exists regarding the optimal implementation strategy, including the selection of radiation delivery techniques and beam energy levels.

Various irradiation techniques for lung SBRT delivery have been reported, including three‐dimensional conformal radiation therapy (3D‐CRT), dynamic conformal arc therapy (DCAT), static intensity‐modulated radiotherapy (IMRT), and volumetric‐modulated arc therapy (VMAT).^9^ Of those, IMRT and VMAT were two common modalities to perform SBRT^10^ and both have been demonstrated to be more conformal than 3D‐CRT and DCAT.^9^ Several studies revealed that VMAT could provide better delivery accuracy and shorter treatment time compared to IMRT in the SBRT of cervical cancer and spine tumor.^11^, ^12^ For the radiotherapy of early‐stage lung cancer using SBRT, coplanar VMAT plan achieved a better plan quality and lower skin dose levels than those with coplanar IMRT plan and decreased delivery time at most by 70%.^13^ However, VMAT usually irradiates the tumor from more incident angles, which may increase the volume of the lung receiving low‐dose radiation.^13^ To our knowledge, several studies comparing VMAT and IMRT in lung cancer have yielded conflicting results, such as the *V*_5Gy_ of whole lung.^13^, ^14^ In practice, considering the diversity of dose and irradiated volume of lung tissue with different techniques, the choice of optimal radiation regimens still be controversial in lung SBRT plans.

Another aspect not often addressed in SBRT plan studies is the photon energy level. Although higher energy photons have the advantage of a lower attenuation with depth, they can also lead to an increased risk of secondary malignancies owing to the presence of neutrons generated in the accelerator head.^15^, ^16^ Protocol‐0915 of Radiation Therapy Oncology Group (RTOG) has recommended the use of photons of energy in the range from 4‐ to 12‐ MV for NSCLC radiotherapy.^17^ However, the issues still existed about the selection of energy levels in lung SBRT plans. Some small lung lesions treated with SBRT are more likely to be eccentric. Considering the single large dose of SBRT, in order to reduce the dose of locally normal tissues, such as skin, the radiation angle needs to be distributed in a wider range, which would generate some beam angles with longer incident distance. In the circumstances, high energy performs its advantage of stronger penetration. However, low energy has advantage on reducing the dose deposited to the adjacent critical organs because of steep dose fall‐off with small penumbra at the field edge.^18^ Moreover, the effect of beam energy level on plan quality was related to the number of beams.^19^ VMAT has a greater number of beam angles than fixed‐field IMRT. Therefore, it needs to be further clarified if the influence of energy on the plan quality of VMAT was less significant than IMRT, making this influence less important.

In addition, rapid dose fall‐off gradients beyond the target are very critical for SBRT, because tissues exposed to high fraction doses are prone to significant dysfunction.^20^ Given the distinction of plan optimization strategies between SBRT and conventional fractionation regimens, it is still unclear about the optimal combination of different techniques and energy levels in lung SBRT. In the present study, in order to demonstrate if an ideal radiation technique may work to best execute SBRT, we used a completely automatic SBRT planning (ASP) system to generate treatment plans, minimizing the impact caused by manual factors, including the experience and clinical preferences, which have been shown to correlate with the quality of the plan.^21^, ^22^, ^23^ And then, we compared the use of 6‐MV photon to 10‐MV photon for all techniques. As far as we know, this is the first study to analyze the treatment plan difference between VMAT and IMRT in SBRT based on automatic planning.

## 2 MATERIALS AND METHODS

### 2.1 Patient selection

Thirty patients with primary or metastatic lung tumors who had received SBRT at our hospital between 2017 and 2019 were selected randomly for analysis. The Clinical Research Committee of the study institute approved the protocol, and the need for written informed consent was waived by the Institutional Review Board.

### 2.2 Treatment plans

The localization, simulation, immobilization, targets and organs at risk (OARs) volumes delineation, and prescription dose constraints were performed according to the RTOG‐0915 protocol.^17^ We used an ASP system, which has been described and validated previously,^24^ to generate the planning auxiliary structures, add beams and initial objectives and constraints, and modulate the parameters and optimization on the basis of the RayStation treatment planning system (RaySearch Laboratories, v4.7). There were three steps in the ASP optimization scheme: initial optimization (Step 1), main optimization (Step 2), and final optimization (Step 3). The Step 1, initial optimization was performed automatically. After evaluating the rationality of the initial optimization objectives of auxiliary structures and OARs, the ASP modified the parameters that contradict the target objectives automatically. Assessment criteria: an objective value of less than 0.0001 (equal to the tolerance) for any OAR was considered reasonable. Step 2, the main optimization consisted of repeated multiple optimizations and parameter adjustments. After each optimization, the planning target volume (PTV) dose was scaled to the prescribed dose and the objective values were computed. Then the dose objectives of auxiliary structures and OARs were adjusted to keep the corresponding objective values within the prespecified range. In the final step, the PTV and OARs were checked according to the prescribed dose and the constraints of OARs. If the PTV coverage, dose fall‐off beyond the target, and OARs sparing met the dose requirements, ASP automatically saved the plan and exited. Otherwise, the projects that did not meet the standards were adjusted and the plan was re‐optimized until all the dose requirements were met or the maximum number of iterations was reached. The SBRT plans of each patient were replanned based on VMAT and IMRT techniques and customized to the accelerator (Elekta Versa HD, Elekta Oncology, UK) with either 6‐ or 10‐MV photon beams. In all, a total of four treatment plans were recreated for each patient: VMAT_6‐MV_, VMAT_10‐MV_, IMRT_6‐MV_, and IMRT_10‐MV_. Collapsed cone algorithm was used to compute the final dose for all plans.

Considering that the higher the number of beams, the longer the treatment time for IMRT, and the fractional displacement increased with the increase of treatment time,^25^ all IMRT plans were created using seven isometric coplanar static fields and optimized by direct machine parameter optimization. Additional parameters were as follows: the minimum segment area was 4 cm^2^; the monitor unit of minimum segment was 5; the maximum number of segments was 50. VMAT plans were generated using two coplanar full arcs. Other parameters were in accordance with those of the IMRT plan.

### 2.3 Dose prescription and plan acceptance criteria

The prescription dose to PTV was 48 Gy in four fractions. According to the protocol of RTOG‐0915, 99% of the PTV receives a minimum of 90% of the prescribed dose and 95% of the PTV is conformally covered by the prescription isodose surface.

The heterogeneity index (*HI*) was calculated in‐line with the International Commission on Radiation Units and Measurements report 83,^26^ and the conformity index (*CI*) was calculated according to the equation of the Paddick index.^27^ *D*_2cm_ was the maximum dose (in % of dose prescribed) to any point 2 cm away from the PTV in any direction; *R*_50%_ was the ratio of 50% prescription isodose volume to PTV. Ten rings (5‐mm width for each ring) outside the PTV were created to limit the dose of normal tissue and evaluate the dose fall‐off.

### 2.4 Statistical analysis

All data were recorded as median value and their interquartile range (25%–75%) for the 30 patients, and dosimetric comparison between plans was performed using the Wilcoxon signed‐ranks test of two related samples. The percentage differences were calculated as follows: (*B *− *A*)/*A* (*A* vs. *B*). The Spearman test was used to estimate the correlation between the percentage differences of *D*_max_ of rings among different plans and the distance away from PTV. Differences were considered significant if a *p* value was <0.05.

## 3 RESULTS

For all 30 patients, clinically acceptable plans could be achieved for VMAT_6‐MV_, VMAT_10‐MV_, IMRT_6‐MV_, and IMRT_10‐MV_. Tumor characteristics are summarized in Table 1. The absolute plan parameters are summarized in Table 2.

**TABLE 1:** Baseline information of all 30 patients in the present study

Variable	*N*	%
Age (years)		
Median (range)	53 (36–80)	
Gender		
Male	23	76.7
Female	7	23.3
Histoloy		
Primary	15	50.0
Metastatic	15	50.0
Site		
Right	13	43.3
Left	17	56.7
PTV Volume (cm^3^)		
Median (range)	18.21 (9.48–29)	

Abbreviation: PTV, planning target volume.

**TABLE 2:** Summary of plan parameters

	**VMAT_6‐MV_**	**VMAT_10‐MV_**	**IMRT_6‐MV_**	**IMRT_10‐MV_**
	Median (25%–75%)	Median (25%–75%)	Median (25%–75%)	Median (25%–75%)
PTV				
*D*_2%_ (Gy)	72.75 (68.48–76.92)	72.72 (70.37–76.14)	68.65 (64.27–71.53)	69.29 (65.91–71.95)
*D*_50%_ (Gy)	57.94 (57.23–58.73)	58.14 (57.75–58.81)	56.54 (55.72–57.07)	56.68 (55.84–57.31)
*D*_95%_ (Gy)	48.00 (47.99–48.03)	48.00 (47.99–48.01)	48.00 (47.99–48.03)	48.00 (47.99–48.03)
*D*_98%_ (Gy)	46.34 (46.04–46.55)	46.24 (46.07–46.40)	46.38 (46.21–46.56)	46.36 (46.10–46.57)
*HI*	0.45 (0.39–0.53)	0.45 (0.41–0.52)	0.39 (0.32–0.45)	0.40 (0.35–0.45)
*CI*	0.86 (0.82–0.88)	0.86 (0.83–0.88)	0.85 (0.82–0.88)	0.85 (0.82–0.87)
MUs	2945.18 (2696–3117.35)	3003.34 (2709.75–3274.06)	3020.12 (2823.58–3234.61)	2979.29 (2749.64–3329.84)
Spinal cord				
*D*_max_ (Gy)	6.16 (4.10–9.74)	6.43 (4.71–9.67)	9.94 (6.60–13.35)	11.37 (6.83–14.26)
*D* ${}_{1.2\;{\mathrm{cm}}^{3}}$ (Gy)	4.96 (3.54–8.18)	5.21 (4.01–7.90)	7.33 (1.79–9.83)	7.83 (1.95–10.72)
*D* ${}_{0.35\;{\mathrm{cm}}^{3}}$ (Gy)	5.36 (3.72–8.70)	5.56 (4.20–8.44)	8.44 (3.54–10.51)	9.08 (3.74–11.44)
Esophagus				
*D*_max_ (Gy)	5.61 (4.58–8.33)	5.99 (4.40–9.24)	8.95 (6.85–11.25)	9.54 (7.20–11.87)
*D* ${}_{5\;{\mathrm{cm}}^{3}}$ (Gy)	0.69 (0.28–1.59)	0.89 (0.34–2.33)	0.63 (0.36–2.62)	1.01 (0.46–2.48)
Heart				
*D*_max_ (Gy)	11.23 (7.95–17.79)	11.67 (8.46–19.31)	15.74 (12.92–22.10)	15.60 (13.61–22.38)
*D* ${}_{15\;{\mathrm{cm}}^{3}}$ (Gy)	7.80 (4.91–10.37)	8.00 (5.24–11.03)	11.25 (8.42–13.69)	11.86 (9.65–14.88)
Rib				
*D*_max_ (Gy)	46.97 (24.10–56.94)	47.05 (25.83–57.18)	47.07 (26.16–57.89)	46.71 (28.12–57.45)
*D* ${}_{1\;{\mathrm{cm}}^{3}}$ (Gy)	32.44 (16.57–45.54)	31.95 (16.99–45.42)	32.92 (18.87–45.69)	32.50 (20.07–46.14)
*D* ${}_{2\;{\mathrm{cm}}^{3}}$ (Gy)	29.45 (15.86–41.85)	29.49 (16.31–41.95)	29.55 (17.99–42.40)	29.30 (19.12–43.06)
*D* ${}_{5\;{\mathrm{cm}}^{3}}$ (Gy)	24.27 (13.49–34.32)	25.31 (14.48–34.06)	24.65 (13.83–34.51)	25.32 (14.82–35.09)
*V*_30Gy_ (%)	2 (0–8)	2 (0–8)	3 (0–9)	3 (0–9)
Skin				
*D*_max_ (Gy)	16.63 (12.85–21.65)	16.36 (12.79–21.20)	25.30 (23.34–29.93)	24.02 (21.00–28.13)
*D* ${}_{10\;{\mathrm{cm}}^{3}}$ (Gy)	9.72 (7.90–11.37)	9.83 (7.91–11.62)	13.49 (12.08–15.74)	13.07 (12.08–15.00)
Lung (right and left)				
*D*_mean_ (Gy)	2.24 (1.66–3.92)	2.36 (1.72–4.21)	2.47 (1.95–4.22)	2.60 (2.07–4.51)
*V*_5Gy_ (%)	11 (9–19)	12 (9–20)	13 (11–23)	14 (12–24)
*V*_10Gy_ (%)	6 (4–11)	6 (4–12)	7 (5–14)	8 (5–14)
*V*_20Gy_ (%)	2 (1–5)	2 (1–5)	2 (2–5)	2 (2–6)
Great vessels				
*D*_max_ (Gy)	10.20 (6.91–15.14)	10.79 (7.22–17.32)	12.71 (10.50–17.94)	13.78 (10.95–19.37)
TPBT				
*D*_max_ (Gy)	7.38 (2.53–13.60)	7.51 (3.90–14.00)	8.95 (3.36–14.86)	9.92 (4.54–15.82)
*D* ${}_{4\;{\mathrm{cm}}^{3}}$ (Gy)	3.76 (0.46–7.22)	3.97 (0.65–7.82)	6.64 (0.56–9.23)	6.85 (0.74–10.17)

Abbreviations: *CI*, conformity index; *D*_max_, maximal dose; *D*_mean_, mean dose; $D_{N\;{\mathrm{cm}}^{3}}$, minimum absorbed dose that covers $N\;{\mathrm{cm}}^{3}$ of the volume; *D*_V_, absorbed dose that covers a specified fractional volume *V; HI*, homogeneity index; MUs, monitor units; PTV, planning target volume; TPBT, trachea and proximal bronchial tree; *V*_D_, volume that receives at least the absorbed dose *D *Gy.

### 3.1 Comparison of different techniques with the same energy level (IMRT_6‐MV_ vs. VMAT_6‐MV_ and IMRT_10‐MV_ vs. VMAT_10‐MV_)

As shown in Table 3, VMAT_6/10‐MV_ plans increased *D*_2%_, *D*_50%_ of PTV by 2.47%–5.98% compared to IMRT_6/10‐MV_ plans, whereas the *D*_98%_ of VMAT_10‐MV_ was 0.28% lower than that of IMRT_10‐MV_. Regarding the difference between *CI* and *HI*, VMAT_6/10‐MV_ showed higher *CI* and *HI* values compared to IMRT_6/10‐MV_, and the percentage differences were 0.57%–0.75%, 13.09%–15.25%, respectively. When comparing the *D*_max_ of OARs, VMAT_6/10‐MV_ was lower than IMRT_6/10‐MV_ for almost all OARs, including spinal cord, esophagus, heart, skin, great vessels, and trachea and proximal bronchial tree (TPBT) with percentage differences ranging from 18.32% to 47.95%. VMAT_6/10‐MV_ obviously decreased *D*_mean_, *V*_5Gy_, *V*_10Gy_, and *V*_20Gy_ of whole lung by 9.68%–20.92% than IMRT_6/10‐MV_. However, the *D*_max_ comparison of ribs yielded no significant differences.

**TABLE 3:** Percentage difference in plan parameters between different techniques with the same energy level

	**VMAT_6‐MV_** versus **IMRT_6‐MV_**		**VMAT_10‐MV_** versus **IMRT_10‐MV_**	
	Median (25%–75%)	*P*	Median (25%–75%)	*P*
PTV				
*D*_2%_	–5.98% (−9.61%–2.67%)	0.000	−5.22% (−9.28%–1.84%)	0.000
*D*_50%_	−2.59% (−3.63%–1.85%)	0.000	−2.47% (−3.85%–1.53%)	0.000
*D*_95%_	0.00% (−0.03%–0.00%)	0.491	0.00% (0.00%–0.02%)	0.271
*D*_98%_	0.12% (−0.14%–0.48%)	0.075	0.28% (−0.19%–0.58%)	0.018
*HI*	−15.25% (−24.43%–7.44%)	0.000	−13.09% (−23.20%–4.05%)	0.000
*CI*	−0.57% (−0.94%–0.28%)	0.022	−0.75% (−1.10%–0.42%)	0.000
MUs	3.69% (−7.49%–8.32%)	0.766	1.53% (−12.78%–12.40%)	0.586
Spinal cord				
*D*_max_	30.57% (5.54%–80.87%)	0.000	33.20% (9.21%–70.90%)	0.000
*D* ${}_{1.2\;{\mathrm{cm}}^{3}}$	17.19% (−59.27%–53.48%)	0.090	19.02% (−64.35%–55.42%)	0.075
*D* ${}_{0.35\;{\mathrm{cm}}^{3}}$	16.47% (−31.37%–68.34%)	0.012	18.93% (−29.73%–62.35%)	0.012
Esophagus				
*D*_max_	34.27% (8.87%–72.79%)	0.000	34.19% (14.38%–73.89%)	0.000
*D* ${}_{5\;{\mathrm{cm}}^{3}}$	25.03% (−0.75%–37.70%)	0.006	24.92% (7.60%–46.74%)	0.008
Heart				
*D*_max_	34.50% (10.96%–62.75%)	0.000	31.48% (12.79%–56.39%)	0.000
*D* ${}_{15\;{\mathrm{cm}}^{3}}$	35.07% (10.49%–59.33%)	0.000	37.58% (12.16%–62.59%)	0.000
Rib				
*D*_max_	1.95% (−2.42%–9.41%)	0.229	1.09% (−1.77%–7.88%)	0.141
*D* ${}_{1\;{\mathrm{cm}}^{3}}$	4.66% (−0.06%–13.48%)	0.003	2.52% (1.24%–14.63%)	0.000
*D* ${}_{2\;{\mathrm{cm}}^{3}}$	2.70% (1.09%–10.80%)	0.001	2.62% (1.40%–13.24%)	0.000
*D* ${}_{5\;{\mathrm{cm}}^{3}}$	2.81% (0.64%–7.04%)	0.000	2.53% (0.65%–5.71%)	0.000
*V*_30Gy_	6.28% (−3.77%–30.31%)	0.372	6.62% (−0.27%–14.52%)	0.008
Skin				
*D*_max_	47.95% (13.37%–99.31%)	0.000	31.86% (14.58%–86.97%)	0.000
*D* ${}_{10\;{\mathrm{cm}}^{3}}$	42.23% (30.98%–56.89%)	0.000	36.36% (21.25%–51.26%)	0.000
Lung (right and left)				
*D*_mean_	9.68% (6.44%–14.00%)	0.000	9.73% (7.46%–16.59%)	0.000
*V*_5Gy_	19.74% (13.78%–30.75%)	0.000	20.92% (12.71%–31.28%)	0.000
*V*_10Gy_	18.82% (9.96%–30.02%)	0.000	17.62% (11.20%–31.92%)	0.000
*V*_20Gy_	12.98% (7.68%–18.29%)	0.000	12.77% (8.50%–21.95%)	0.000
Great vessels				
*D*_max_	29.38% (9.56%–66.65%)	0.000	25.35% (7.18%–60.80%)	0.000
TPBT				
*D*_max_	18.32% (9.36%–27.98%)	0.000	22.34% (3.20%–34.95%)	0.000
*D* ${}_{4\;{\mathrm{cm}}^{3}}$	28.23% (13.16%–69.54%)	0.000	30.10% (17.86%–73.03%)	0.000

*Note*: Percentage differences were calculated as (IMRT − VMAT)/VMAT.

Abbreviations: *CI*, conformity index; *D*_max_, maximal dose; *D*_mean_, mean dose; $D_{N\;{\mathrm{cm}}^{3}}$, minimum absorbed dose that covers *N* ${\mathrm{cm}}^{3}$ of the volume; *D*_V_, absorbed dose that covers a specified fractional volume *V; HI*, homogeneity index; MUs, monitor units; PTV, planning target volume; TPBT, trachea and proximal bronchial tree; *V*_D_, volume that receives at least the absorbed dose *D* Gy.

### 3.2 Comparison of different techniques with different energy levels (IMRT_10‐MV_ vs. VMAT_6‐MV_ and IMRT_6‐MV_ vs. VMAT_10‐MV_)

Similar results were found when comparing different techniques with different energy levels (Table 4). VMAT_6/10‐MV_ could significantly increase *D*_2%_, *D*_50%_ by 2.37%–6.09% but was comparable for *D*_95%_ compared to IMRT_10/6‐MV_. *CI* and *HI* of PTV were increased by 0.81% and 14.83% using VMAT technique. When comparing OARs, VMAT showed advantages in *D*_max_ reduction of spinal cord, esophagus, heart, skin, great vessels, and TPBT with percentage differences ranging from 14.09% to 45.13%, excepting ribs. Moreover, *D*_mean_, *V*_5Gy_, *V*_10Gy_, and *V*_20Gy_ of whole lung for VMAT were significantly decreased by 5.56%–26.11% compared to those parameters for IMRT.

**TABLE 4:** Percentage difference in plan parameters between different techniques with different energy levels

	**VMAT_6‐MV_** versus **IMRT_10‐MV_**		**VMAT_10‐MV_** versus **IMRT_6‐MV_**	
	Median (25%–75%)	*P*	Median (25%–75%)	*P*
PTV				
*D*_2%_	−4.66% (−11.63%–1.88%)	0.000	−6.09% (−9.30%–2.65%)	0.000
*D*_50%_	−2.37% (−3.67%–1.15%)	0.000	−2.84% (−3.97%–1.86%)	0.000
*D*_95%_	0.00% (−0.01%–0.02%)	0.861	0.00% (−0.02%–0.00%)	0.734
*D*_98%_	0.06% (−0.26%–0.39%)	0.491	0.42% (−0.09%–0.81%)	0.001
*HI*	−11.75% (−25.09%–3.91%)	0.001	−14.83% (−21.51%–7.39%)	0.000
*CI*	−0.54% (−0.91%–0.07%)	0.004	−0.81% (−1.21%–0.24%)	0.000
MUs	2.42% (−8.66%–8.78%)	0.894	0.90% (−11.97%–10.36%)	0.673
Spinal cord				
*D*_max_	43.11% (8.30%–102.04%)	0.000	24.83% (6.95%–52.08%)	0.001
*D* ${}_{1.2\;{\mathrm{cm}}^{3}}$	17.17% (−58.49%–68.11%)	0.028	13.18% (−61.32%–47.67%)	0.237
*D* ${}_{0.35\;{\mathrm{cm}}^{3}}$	14.85% (−18.42%–88.71%)	0.004	14.84% (−37.91%–47.62%)	0.039
Esophagus				
*D*_max_	43.33% (20.95%–81.55%)	0.000	23.27% (3.82%–70.38%)	0.000
*D* ${}_{5\;{\mathrm{cm}}^{3}}$	47.07% (26.05%–82.55%)	0.000	7.04% (−26.13%–30.56%)	0.465
Heart				
*D*_max_	35.93% (10.87%–54.87%)	0.000	30.04% (10.03%–62.05%)	0.000
*D* ${}_{15\;{\mathrm{cm}}^{3}}$	48.02% (21.89%–91.48%)	0.000	24.74% (4.86%–43.26%)	0.000
Rib				
*D*_max_	1.29% (−0.30%–12.62%)	0.057	1.06% (−1.53%–4.54%)	0.371
*D* ${}_{1\;{\mathrm{cm}}^{3}}$	4.45% (0.38%–18.22%)	0.000	2.25% (0.26%–7.85%)	0.002
*D* ${}_{2\;{\mathrm{cm}}^{3}}$	4.95% (1.36%–15.84%)	0.000	1.41% (0.31%–7.25%)	0.009
*D* ${}_{5\;{\mathrm{cm}}^{3}}$	5.29% (2.14%–14.56%)	0.000	0.35% (−2.08%–4.22%)	0.371
*V*_30Gy_	14.93% (2.46%–53.93%)	0.006	−2.98% (−8.59%–12.59%)	0.338
Skin				
*D*_max_	35.09% (12.71%–86.14%)	0.000	45.13% (18.64%–100.11%)	0.000
*D* ${}_{10\;{\mathrm{cm}}^{3}}$	39.35% (21.71%–56.94%)	0.000	42.82% (27.19%–53.07%)	0.000
Lung (right and left)				
*D*_mean_	16.49% (12.26%–19.96%)	0.000	5.56% (1.97%–10.11%)	0.000
*V*_5Gy_	26.11% (17.42%–37.54%)	0.000	14.28% (8.21%–26.30%)	0.000
*V*_10Gy_	20.86% (15.04%–36.31%)	0.000	13.71% (6.02%–28.12%)	0.000
*V*_20Gy_	17.38% (12.80%–24.86%)	0.000	9.30% (3.22%–16.57%)	0.000
Great vessels				
*D*_max_	32.43% (24.06%–69.41%)	0.000	19.17% (1.98%–64.15%)	0.000
TPBT				
*D*_max_	34.10% (14.96%–62.40%)	0.000	14.09% (−11.83%–19.35%)	0.024
*D* ${}_{4\;{\mathrm{cm}}^{3}}$	44.48% (21.51%–78.42%)	0.000	22.35% (6.01%–65.57%)	0.000

*Note*: Percentage differences were calculated as (IMRT − VMAT)/VMAT.

Abbreviations: *CI*, conformity index; *D*_max_, maximal dose; *D*_mean_, mean dose; $D_{N\;{\mathrm{cm}}^{3}}$, minimum absorbed dose that covers *N* ${\mathrm{cm}}^{3}$ of the volume; *D*_V_, absorbed dose that covers a specified fractional volume *V; HI*, homogeneity index; MUs, monitor units; PTV, planning target volume; TPBT, trachea and proximal bronchial tree; *V*_D_, volume that receives at least the absorbed dose *D *Gy.

### 3.3 Comparison of different energy levels with the same techniques (IMRT_6‐MV_ vs. IMRT_10‐MV_ and VMAT_6‐MV_ vs. VMAT_10‐MV_)

As shown in Table 5, the differences in the *D*_2%_, *D*_98%_, *HI*, and *CI* between 6‐ and 10‐MV plans were not statistically significant respectively. Considering the dose sparing of OARs, 6‐MV plans performed 4.68%–8.91% lower level of *D*_max_ of spinal cord, esophagus, great vessels, and TPBT than 10‐MV plans. Similarly, *D*_mean_, *V*_5Gy_, *V*_10Gy_, and *V*_20Gy_ of whole lung were also reduced by 2.79%–5.25% using 6‐MV.

**TABLE 5:** Percentage difference in plan parameters between different energy levels with the same techniques

	**VMAT_6‐MV_** versus **VMAT_10‐MV_**		**IMRT_6‐MV_** versus **IMRT_10‐MV_**	
	Median (25%–75%)	*P*	Median (25%–75%)	*P*
PTV				
*D*_2%_	0.42% (−1.55%–2.34%)	0.558	0.97% (−1.35%–2.99%)	0.382
*D*_50%_	0.15% (−0.14%–1.09%)	0.047	0.16% (−0.27%–0.65%)	0.199
*D*_95%_	0.00% (−0.02%–0.00%)	0.185	0.00% (0.00%–0.04%)	0.530
*D*_98%_	−0.27% (−0.52%–0.15%)	0.090	−0.14% (−0.35%–0.13%)	0.090
*HI*	1.30% (−5.01%–5.92%)	0.600	3.26% (−4.10%–10.85%)	0.393
*CI*	0.10% (−0.21%–0.62%)	0.102	0.02% (−0.67%–0.45%)	0.704
MUs	0.88% (−2.16%–4.27%)	0.329	−0.39% (−3.27%–2.35%)	0.491
Spinal cord				
*D*_max_	6.93% (2.33%–17.84%)	0.000	4.68% (0.47%–12.14%)	0.000
*D* ${}_{1.2\;{\mathrm{cm}}^{3}}$	7.58% (−1.61%–18.35%)	0.009	7.21% (3.72%–11.36%)	0.000
*D* ${}_{0.35\;{\mathrm{cm}}^{3}}$	8.63% (−1.01%–18.08%)	0.008	5.92% (−0.01%–12.22%)	0.002
Esophagus				
*D*_max_	5.41% (−0.59%–11.59%)	0.006	6.17% (−0.16%–10.80%)	0.001
*D* ${}_{5\;{\mathrm{cm}}^{3}}$	19.28% (7.41%–38.85%)	0.000	23.05% (6.90%–40.72%)	0.000
Heart				
*D*_max_	0.39% (−2.22%–7.95%)	0.280	0.05% (−3.89%–5.48%)	0.926
*D* ${}_{15\;{\mathrm{cm}}^{3}}$	5.97% (2.13%–9.91%)	0.001	8.46% (3.70%–11.37%)	0.000
Rib				
*D*_max_	1.11% (−1.17%–5.23%)	0.098	1.42% (−0.59%–3.73%)	0.045
*D* ${}_{1\;{\mathrm{cm}}^{3}}$	1.76% (−0.81%–4.79%)	0.066	1.66% (0.21%–3.81%)	0.000
*D* ${}_{2\;{\mathrm{cm}}^{3}}$	1.30% (−0.57%–4.53%)	0.032	1.73% (0.29%–4.28%)	0.000
*D* $5\;{\mathrm{cm}}^{3}$	3.30% (−0.77%–7.23%)	0.007	2.15% (0.08%–4.85%)	0.000
*V*_30Gy_	4.03% (−2.30%–27.98%)	0.123	8.56% (0.65%–16.69%)	0.001
Skin				
*D*_max_	−0.64% (−2.56%–3.68%)	0.658	−6.25% (−13.39%–1.20%)	0.000
*D* ${}_{10\;{\mathrm{cm}}^{3}}$	2.53% (−0.46%–5.89%)	0.005	−1.99% (−6.77%–1.34%)	0.012
Lung (right and left)				
*D*_mean_	4.52% (3.17%–5.92%)	0.000	5.25% (3.76%–7.09%)	0.000
*V*_5Gy_	4.02% (1.92%–6.11%)	0.000	3.99% (3.15%–5.91%)	0.000
*V*_10Gy_	2.79% (0.00%–6.96%)	0.002	4.71% (2.33%–5.99%)	0.000
*V*_20Gy_	3.18% (−1.03%–5.42%)	0.003	4.06% (0.88%–7.40%)	0.000
Great vessels				
*D*_max_	4.82% (−0.24%–13.04%)	0.005	6.26% (−0.23%–9.38%)	0.001
TPBT				
*D*_max_	8.91% (−1.77%–39.86%)	0.028	7.04% (3.52%–32.70%)	0.000
*D* ${}_{4\;{\mathrm{cm}}^{3}}$	6.91% (2.77%–18.59%)	0.000	7.13% (2.45%–14.17%)	0.000

*Note*: Percentage differences were calculated as (10‐MV − 6‐MV)/6‐MV.

Abbreviations: *CI*, conformity index; *D*_max_, maximal dose; *D*_mean_, mean dose; $D_{N\;{\mathrm{cm}}^{3}}$, minimum absorbed dose that covers *N* ${\mathrm{cm}}^{3}$ of the volume; *D*_V_, absorbed dose that covers a specified fractional volume *V; HI*, homogeneity index; MUs, monitor units; PTV, planning target volume; TPBT, trachea and proximal bronchial tree; *V*_D_, volume that receives at least the absorbed dose *D *Gy.

### 3.4 Comparison of PTV dose fall‐off

**FIGURE 1:**
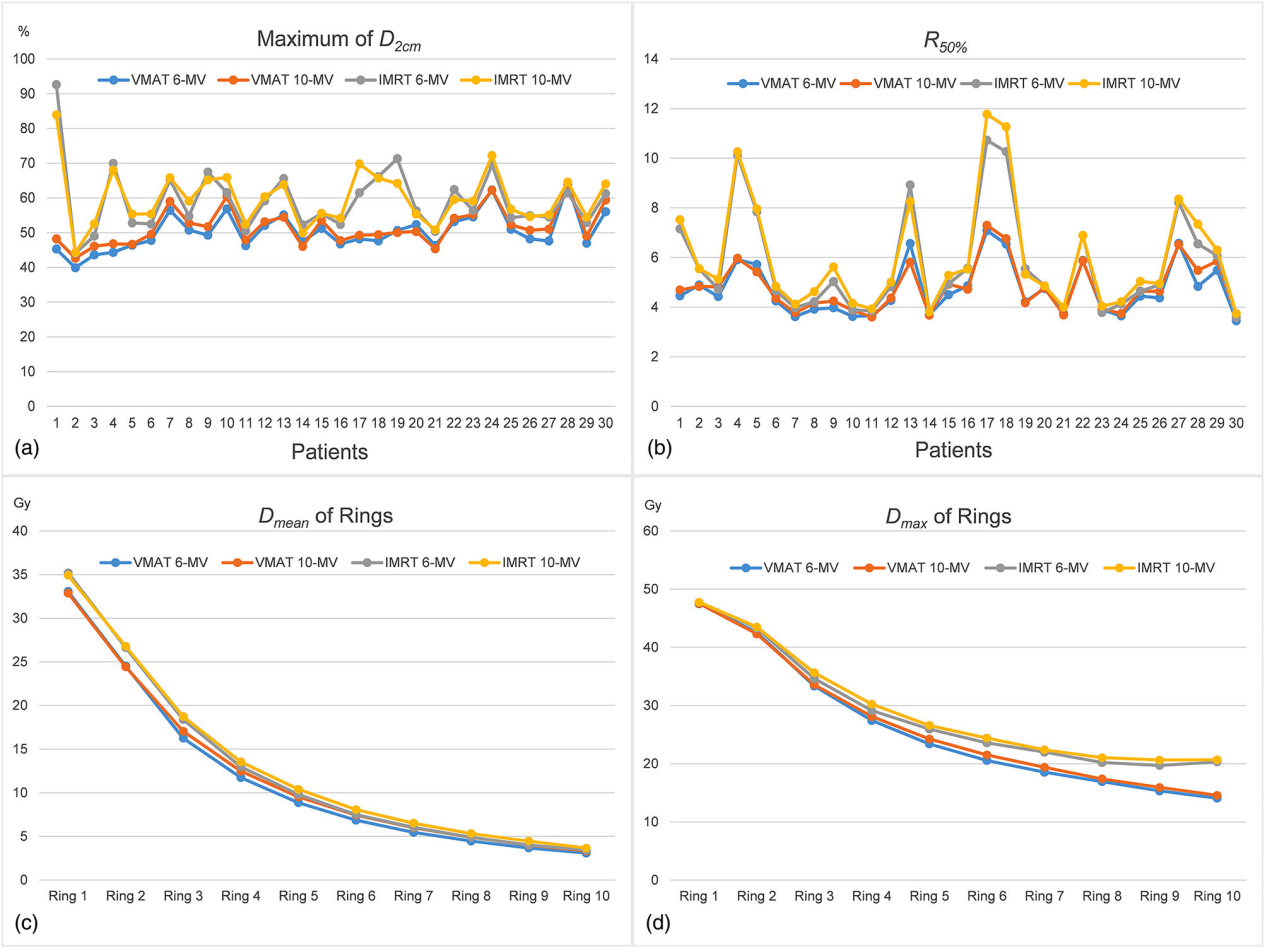
Dose fall‐off analysis among four stereotactic body radiation therapy (SBRT) treatment plans: (a) the maximum of *D*_2cm_ of all 30 patients; (b) *R*_50%_ of all 30 patients; (c) *D*_mean_ (median value) of 10 rings; and (d) *D*_max_ (median value) of 10 rings

**FIGURE 2:**
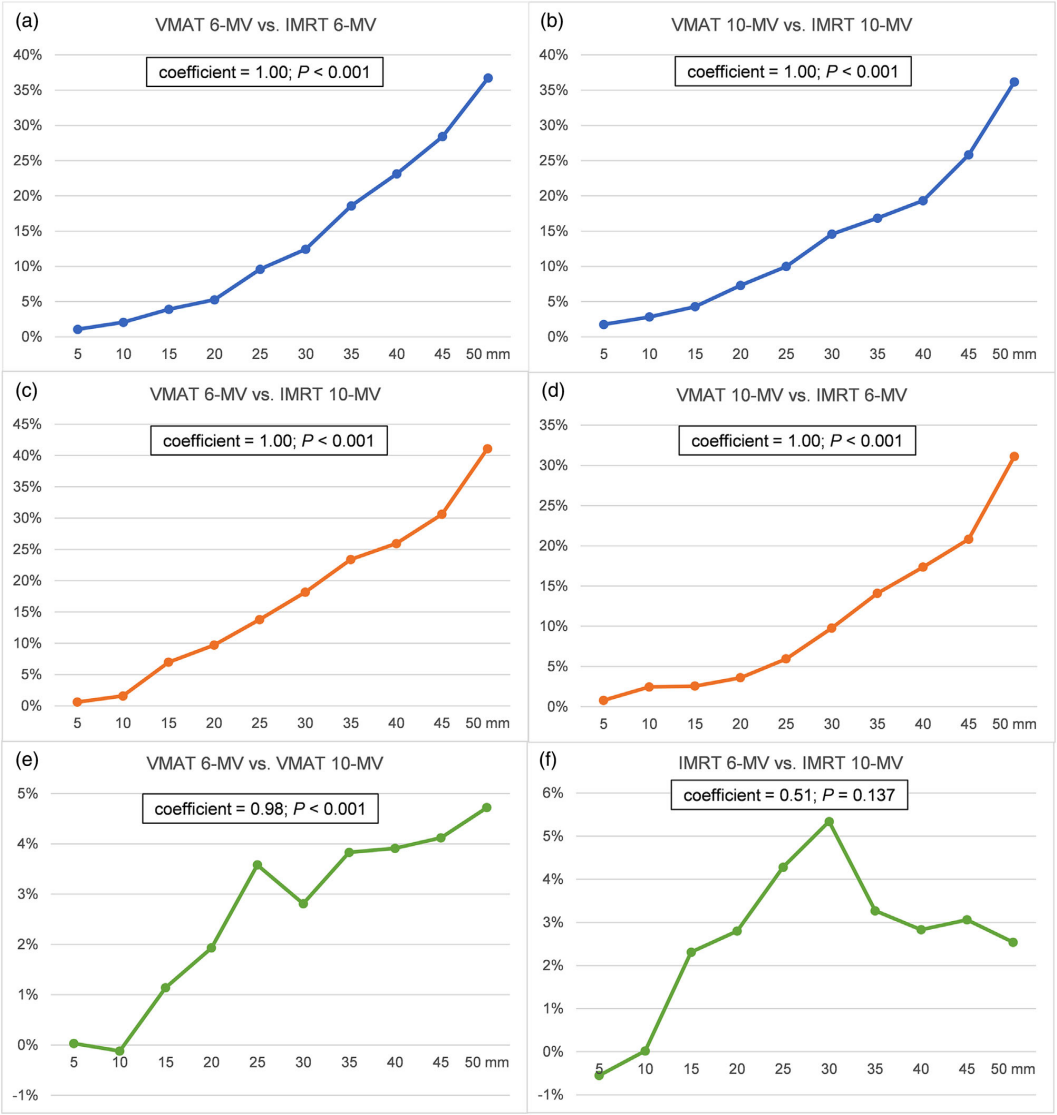
Correlation analysis of percentage differences of *D*_max_ of rings and distance away from planning target volume (PTV): (a) volumetric‐modulated arc therapy (VMAT)_6‐MV_ versus intensity‐modulated radiotherapy (IMRT)_6‐MV_; (b) VMAT_10‐MV_ versus IMRT_10‐MV_; (c) VMAT_6‐MV_ versus IMRT_10‐MV_; (d) VMAT_10‐MV_ versus IMRT_6‐MV_

Figure 1a,b shows the maximum of *D*_2cm_ and *R*_50%_ for each of the 30 patients. The maximum of *D*_2cm_ of IMRT_6/10‐MV_ were obviously higher than those of VMAT_6/10‐MV_. The same trend was visible for the *R*_50%_ with IMRT_6/10‐MV_ being higher than VMAT_6/10‐MV_. *D*_2cm_ and *R*_50%_ for almost all patients in VMAT met the requirements of RTOG‐0915, whereas IMRT did not. The observed values of both parameters were the lowest for VMAT_6‐MV_ and the highest for IMRT_10‐MV_. Dose fall‐off curve based on *D*_mean_ and *D*_max_ of 10 rings out of PTV are presented in Figure 1c,d. Results showed that the radiation dose fall‐off curve was steeper for VMAT plans than IMRT plans regardless of the energy used (6‐ or 10‐MV). Notably, *D*_mean_ of rings decreased dramatically from Ring 1 to 3 and then moderately from Ring 4 to 10 for all treatment plans. Considering *D*_max_ of Rings, VMAT plans and IMRT plans showed a similar downward trend until Ring 4, after that, a gap emerged between those two different techniques. The dose fall‐off of VMAT plans was sharper than IMRT plans from Ring 4 to 10. In addition, correlation analysis showed the percentage differences of *D*_max_ of rings between VMAT and IMRT were significantly related to the distance away from PTV (Figure 2). Those differences became more obvious along with the distance further. Moreover, VMAT_6/10‐MV_ decreased the integral dose by 1.45–2.77 Gy (median difference) than IMRT_6/10‐MV_.

## 4 DISCUSSION

The present study qualified dosimetric differences between delivery techniques with either IMRT‐ or VMAT‐based SBRT using 6‐ or 10‐MV beam energy for lung tumors. Importantly, we used the fully automated SBRT plan system to avoid any deviations caused by manual adjustment to the most extent. The automatic planning system has been evaluated in a previous study and shown preferable quality than the manual system.^24^ We found that the differences between different techniques (IMRT vs. VMAT without regard to energy level) were more obvious than those between different energy levels with the same technique (IMRT_6‐MV_ vs. IMRT_10‐MV_, VMAT_6‐MV_ vs. VMAT_10‐MV_). And then, findings revealed that VMAT showed statistically significant improvement in the protection of OARs and dose fall‐off of normal tissues compared to IMRT treatments. Meanwhile, VMAT also performed better conformity but weakening homogeneity. In addition, 6‐MV plans performed the ability to reduce dose to OARs when compared with 10‐MV plans, whereas this improvement was not pronounced in target coverage.

SBRT for early‐stage NSCLC patients delivers higher doses using fewer fractions. Currently, IMRT has been replaced by VMAT in many institutions because of its shorter delivery time.^10^ Rossi et al. evaluated the intrafraction motion in SBRT for NSCLC and found that VMAT‐based SBRT could reduce intrafraction motion and consequently improve the accuracy of dose delivery due to the significant reduction of delivery time compared with IMRT‐based SBRT.^10^ Regarding the PTV conformity calculated by 100% prescription dose, Holt et al. reported there was no significant difference between VMAT and IMRT. However, in our study, VMAT_6/10‐MV_ brought superior conformity than IMRT_6/10‐MV_. Considering the advantage of automatic planning with minimal bias, we strongly believe that VMAT can improve the conformity of lung SBRT plan. Moreover, we found the PTV *HI* of VMAT_6/10‐MV_ was greater than that of IMRT_6/10‐MV_. However, there was not much difference in absolute values and not much influence on clinical practice.

Lung sparing is regarded as an important factor in lung SBRT plan due to radiation‐induced lung toxicity (RILT). Holt et al. analyzed three different SBRT plans of early‐stage lung cancer using coplanar and noncoplanar IMRT and coplanar VMAT (using SmartArc). They found that the treatment plan quality for coplanar VMAT using one dual arc was better than that of coplanar IMRT plans using 9 equidistant coplanar beams with a maximum of 18 segments in total, and the *V*_5Gy_ of whole lung in VMAT had improved significantly compared to that in coplanar IMRT.^13^ However, Jiang et al. found the opposite results in a conventional fractionation lung cancer study. The higher *V*_5Gy_ and *V*_10Gy_ were observed in the single‐/partial‐arc VMAT plans compared to IMRT plans using 5–7 beams with a maximum of 100 segments in total.^14^ One possible explanation is that they adopted different VMAT arc numbers and segment numbers. Another reason might be they used manual planning, which may introduce subjective error and affect the results of comparison. In our study, the parameters, including *MLD*, *V*_5Gy_, *V*_10Gy_, and *V*_20Gy_ of IMRT, were significantly higher than those of VMAT no matter using 6‐ or 10‐MV. Our results may help resolve the confusion caused by the conflicting results of previous studies because all those comparisons were performed based on unbiased ASP and we believed our conclusion was stable. As reported previously, dosimetric parameters, including *MLD, V*_5Gy_, and *V*_20Gy_, were related to RILT,^28^, ^29^, ^30^ which caused by excessive radiation dose to the normal lung tissue and should be noticed in lung SBRT performance.^31^ Therefore, our study robustly and comprehensively confirmed the advantage of VMAT in reducing pulmonary toxicity for lung tumor SBRT.

In addition to whole lung, it is important to reduce the dose to other OARs to minimize the side effects of lung SBRT, including central airways, esophagus, great vessels, heart, skin, and spinal cord. The previous study demonstrated that, when comparing the coplanar VMAT and the coplanar IMRT, the maximal doses to the OARs, including heart, esophagus, and skin, were significantly lower for VMAT while there was no difference in the *D*_max_ of spinal cord and *V*_5Gy_ of chest wall.^13^ However, in our study, the VMAT_6/10‐MV_ were superior to the IMRT_6/10‐MV_ for almost all OARs dose indices, except for the *D*_max_ of rib, and the absolute dose improvement was mostly greater than that in the study of Holt et al.^13^ Considering the comparisons were based on unbiased ASP, we believed our results were stable. Moreover, the *D*_max_ of rib in VMAT was higher than that in IMRT. One possible explanation was that the distance between lesions and chest wall was shorter in peripheral lung tumors, which was mainly discussed in the present study, resulting in the *D*_max_ of ribs approximating the maximum dose of targets.

Previous studies revealed that lateral scatter of high‐energy electrons leads to an extended path in low‐density tissues such as the lung, resulting in the augment of beam penumbra, larger volumes of lung irradiated, and reduced target dose.^32^ Hence, low‐energy photons were suggested for lung tumor radiotherapy to minimize lateral electronic disequilibrium and to decrease the irradiated volume of the normal lung tissue.^33^ Commonly, 6‐MV photon beams are typically used for SBRT due to the small beam penumbra of an open‐field beam compared to other higher photon energies in conventional linear accelerators.^34^ However, with the use of IMRT or VMAT, the effective penumbra realized in the treatment plan may not be so clear due to the impact of beam fluence optimization.^35^ Henry et al. found that in liver SBRT, 10‐MV photon energy using noncoplanar, fixed‐field IMRT delivery technique approach showed a faster dose fall‐off and improved up to 18.5% in the *R*_30%_ parameter compared to 6‐MV, but no difference was noted in the *R*_50%_.^34^ However, the variation of tissue density in lung and liver limited the extension of the conclusion. There were few studies performing dosimetric comparing of SBRT using different energy levels. In the present study, we found that the dose differences of the same technique with different energy levels were slighter. The target coverage was comparable between 6‐ and 10‐MV regardless of techniques.

Because of the high ablative doses used in SBRT, high‐quality SBRT treatment plans should ensure fast dose fall‐off and compact dose distributions. Any improvements in SBRT treatment plan quality by improving the dose fall‐off may have implications for SBRT treatments. A fast dose fall‐off is essential and beneficial to protect normal tissue in lung SBRT. RTOG‐0813 and 0915 recommend using *D*_2cm_ and *R*_50%_ as plan quality metrics for evaluation of normal tissue sparing and dose fall‐off in SBRT of lung lesion. Clearly, the improved compactness from the faster dose fall‐off translates into a reduction of dose in involved and adjacent normal tissues. In the setting of the RTOG‐0915 trial, an *R*_50%_ value was stipulated to be <2.9–7.5; the maximum *D*_2cm_ in any direction criterion was stipulated to be <50.0%–94.0% of the prescription dose. Delgado et al. evaluated the target dose fall‐off in IMRT and VMAT planning techniques for cervical SBRT through analyzing *R*_50%_ and found that VMAT_3‐arc_ 10‐MV appears to provide the best dose fall‐off than IMRT.^11^ However, there have been few research studies comparing the dose fall‐off using VMAT and IMRT treatment plans for lung tumors. In the present study, VMAT offered an obvious reduction of *R*_50%_ compared to IMRT technique, whereas the values of *R*_50%_ using 6‐MV were significantly lower than those using 10‐MV. Similarly, the comparison of *D*_2cm_ also showed the same trend except that the difference between IMRT_6‐MV_ and IMRT_10‐MV_ was not significant. That is, VMAT_6‐MV_ provided a steeper dose fall‐off outside of the target volume in lung SBRT. Notably, the dose fall‐off advantage of VMAT over IMRT is greater than that of 6‐MV over 10‐MV, both in terms of absolute dose and the number of patients meeting the RTOG‐0915 criterion. Therefore, VMAT and 6‐MV are more suitable for lung tumor SBRT than IMRT and 10‐MV.

Normal tissue sparing could be also analyzed using multiple concentric rings outside the PTV, which has been studied among stereotactic radiosurgery plans.^36^ In the present study, we conducted 10 concentric rings evaluating the dose fall‐off of normal tissues and compared the dosimetric parameters inside every ring. As far as we know, this is the study using the most rings in SBRT to date. Consistent with the results for *R*_50%_ and *D*_2cm_, the analysis based on *D*_max_ of rings concluded that VMAT could perform a faster dose fall‐off than IMRT, which further revealed the advantage of VMAT in involved and adjacent normal tissue sparing when treating lung tumors using SBRT. The potential gains in dose fall‐off may allow for a potential dose escalation to the target volume, and more aggressive dose may be associated with higher local control rates of disease.^37^ Moreover, the dose fall‐off curve based *D*_mean_ of rings was steeper from Ring 1 to 4 and then became mild from Ring 4 to 10, although the automatic optimization strategy did not favor either ring. This trend suggested that the dose fall‐off gradient of normal tissues close to PTV was sharper compared to tissues far away from PTV, with an inflection point at 1.5–2.0 cm away from PTV. A discrepant trend was found in the curve of *D*_max_ of rings. There was an obvious separation between VMAT plans and IMRT plans since Ring 4, indicating VMAT techniques provided better protection for normal tissues 2 cm or greater away from PTV. This kind of advantage was enhanced along with the distance further. Moreover, the integral dose is important in assessing SBRT plans because it is able to detect unsuspected regions of a high absorbed dose beyond PTV. We found VMAT_6/10‐MV_ decreased the integral dose compared to IMRT_6/10‐MV_, which could reduce the risk of late effects, such as carcinogenesis.

The limitations of our study should also be aware of. First, it was a retrospective design from a single center, and the risk of selection bias may exist. And then, we compared the OARs sparing that had influences on radiation‐related normal tissue toxicities. However, clinical outcomes, especially SBRT adverse events of patients, were not analyzed in the present study. Definitely, prospective studies exploring optimal choice were needed for the SBRT plans in the future. Despite these limitations, we believe that our report can serve as a hypothesis‐generating study.

## 5 CONCLUSIONS

In the present study, using an automatic planning system, we performed a comprehensive plan comparison of VMAT to IMRT in lung SBRT using either 6‐ or 10‐MV photon energies. The differences between different techniques were more obvious than those between different energy levels with the same technique. VMAT showed prominent benefits in the protection of OARs and provided sharper dose fall‐off of normal tissues than IMRT treatments. For lung SBRT plans, VMAT techniques may be superior to IMRT techniques and 6‐MV energy level was a better choice than 10‐MV.

## CONFLICT OF INTEREST

The authors report no conflict of interest with this study. We declare that we do not have any commercial or associative interest that represents a conflict of interest in connection with this work.

## AUTHOR CONTRIBUTIONS

(I) Conception and design: Jianghong Xiao and Xingchen Peng; (II) administrative support: Jianghong Xiao; (III) provision of study materials or patients: Zhigong Wei and Xingchen Peng; (IV) collection and assembly of data: Zhigong Wei, Xingchen Peng, Ling He, and Zheran Liu; (V) data analysis and interpretation: Zhigong Wei, Xingchen Peng, and Jingjing Wang; (VI) manuscript writing: all authors; and (VII) final approval of manuscript: all authors.

## Data Availability

The data that support the findings of this study are available on request from the corresponding author. The data are not publicly available due to privacy or ethical restrictions.

## References

[1] Potters L , Kavanagh B , Galvin JM , et al. American Society for Therapeutic Radiology and Oncology (ASTRO) and American College of Radiology (ACR) practice guideline for the performance of stereotactic body radiation therapy. Int J Radiat Oncol Biol Phys. 2010;76(2):326‐332. doi:10.1016/j.ijrobp.2009.09.042. Feb 1. 20117285

[2] Benedict SH , Yenice KM , Followill D , et al. Stereotactic body radiation therapy: the report of AAPM Task Group 101. Med Phys. 2010;37(8):4078‐4101. doi:10.1118/1.3438081. Aug. 20879569

[3] Baumann P , Nyman J , Hoyer M , et al. Outcome in a prospective phase II trial of medically inoperable stage I non‐small‐cell lung cancer patients treated with stereotactic body radiotherapy. J Clin Oncol. 2009;27(20):3290‐3296. doi:10.1200/JCO.2008.21.5681. Jul 10. 19414667

[4] Timmerman R , Paulus R , Galvin J , et al. Stereotactic body radiation therapy for inoperable early stage lung cancer. JAMA. 2010;303(11):1070‐1076. doi:10.1001/jama.2010.261. Mar 17. 20233825 PMC2907644

[5] Wulf J , Haedinger U , Oppitz U , Thiele W , Mueller G , Flentje M . Stereotactic radiotherapy for primary lung cancer and pulmonary metastases: a noninvasive treatment approach in medically inoperable patients. Int J Radiat Oncol Biol Phys. 2004;60(1):186‐196. doi:10.1016/j.ijrobp.2004.02.060. Sep 1. 15337555

[6] Timmerman RD , Paulus R , Pass HI , et al. Stereotactic body radiation therapy for operable early‐stage lung cancer: findings from the NRG oncology RTOG 0618 trial. JAMA Oncol. 2018;4(9):1263‐1266. doi:10.1001/jamaoncol.2018.1251. Sep 1. 29852037 PMC6117102

[7] Onishi H , Shirato H , Nagata Y , et al. Stereotactic body radiotherapy (SBRT) for operable stage I non‐small‐cell lung cancer: can SBRT be comparable to surgery?. Int J Radiat Oncol Biol Phys. 2011;81(5):1352‐1358. doi:10.1016/j.ijrobp.2009.07.1751. Dec 1. 20638194

[8] Horner‐Rieber J , Bernhardt D , Blanck O , et al. Long‐term follow‐up and patterns of recurrence of patients with oligometastatic NSCLC treated with pulmonary SBRT. Clin Lung Cancer. 2019;20(6):e667‐e677. doi:10.1016/j.cllc.2019.06.024. Nov. 31327644

[9] Giglioli FR , Clemente S , Esposito M , et al. Frontiers in planning optimization for lung SBRT. Phys Med. 2017;44:163‐170. doi:10.1016/j.ejmp.2017.05.064. Dec. 28566240

[10] Rossi MM , Peulen HM , Belderbos JS , Sonke JJ . Intrafraction motion in stereotactic body radiation therapy for non‐small cell lung cancer: intensity modulated radiation therapy versus volumetric modulated arc therapy. Int J Radiat Oncol Biol Phys. 2016;95(2):835‐843. doi:10.1016/j.ijrobp.2016.01.060. Jun 1. 27131084

[11] Brito Delgado A , Cohen D , Eng TY , et al. Modeling the target dose fall‐off in IMRT and VMAT planning techniques for cervical SBRT. Med Dosim. 2018;43(1):1‐10. doi:10.1016/j.meddos.2017.07.009. Spring. 29223302

[12] Ouyang Z , LaHurd DV , Balagamwala EH , Chao ST , Suh JH , Xia P . Treatment planning of VMAT and step‐and‐shoot IMRT delivery techniques for single fraction spine SBRT: an intercomparative dosimetric analysis and phantom‐based quality assurance measurements. J Appl Clin Med Phys. 2020;21(1):62‐68. doi:10.1002/acm2.12788. Jan. PMC6964769 31821729

[13] Holt A , van Vliet‐Vroegindeweij C , Mans A , Belderbos JS , Damen EM . Volumetric‐modulated arc therapy for stereotactic body radiotherapy of lung tumors: a comparison with intensity‐modulated radiotherapy techniques. Int J Radiat Oncol Biol Phys. 2011;81(5):1560‐1567. doi:10.1016/j.ijrobp.2010.09.014. Dec 1. 21300461

[14] Jiang X , Li T , Liu Y , et al. Planning analysis for locally advanced lung cancer: dosimetric and efficiency comparisons between intensity‐modulated radiotherapy (IMRT), single‐arc/partial‐arc volumetric modulated arc therapy (SA/PA‐VMAT). Radiat Oncol. 2011;6:140. doi:10.1186/1748-717X-6-140. Oct 21. 22014217 PMC3207896

[15] Kry SF , Followill D , White RA , Stovall M , Kuban DA , Salehpour M . Uncertainty of calculated risk estimates for secondary malignancies after radiotherapy. Int J Radiat Oncol Biol Phys. 2007;68(4):1265‐1271. doi:10.1016/j.ijrobp.2007.04.014. Jul 15. 17637398

[16] Kry SF , Salehpour M , Followill DS , et al. The calculated risk of fatal secondary malignancies from intensity‐modulated radiation therapy. Int J Radiat Oncol Biol Phys. 2005;62(4):1195‐1203. doi:10.1016/j.ijrobp.2005.03.053. Jul 15. 15990025

[17] Videtic GM , Paulus R , Singh AK , et al. Long‐term follow‐up on NRG oncology RTOG 0915 (NCCTG N0927): a randomized phase 2 study comparing 2 stereotactic body radiation therapy schedules for medically inoperable patients with stage I peripheral non‐small cell lung cancer. Int J Radiat Oncol Biol Phys. 2019;103(5):1077‐1084. doi:10.1016/j.ijrobp.2018.11.051. Apr 1 30513377 PMC6454873

[18] Zhang Y , Feng Y , Ahmad M , Ming X , Zhou L , Deng J . Intermediate megavoltage photon beams for improved lung cancer treatments. PLoS One. 2015;10(12):e0145117. doi:10.1371/journal.pone.0145117 26672752 PMC4682946

[19] Pirzkall A , Carol MP , Pickett B , Xia P , Roach M , Verhey LJ . The effect of beam energy and number of fields on photon‐based IMRT for deep‐seated targets. Int J Radiat Oncol Biol Phys. 2002;53(2):434‐442. doi:10.1016/s0360-3016(02)02750-5. Jun 1. 12023148

[20] Timmerman RD . An overview of hypofractionation and introduction to this issue of seminars in radiation oncology. Semin Radiat Oncol. 2008;18(4):215‐222. doi:10.1016/j.semradonc.2008.04.001. Oct. 18725106

[21] Bohsung J , Gillis S , Arrans R , et al. IMRT treatment planning: a comparative inter‐system and inter‐centre planning exercise of the ESTRO QUASIMODO group. Radiother Oncol. 2005;76(3):354‐361. doi:10.1016/j.radonc.2005.08.003. Sep. 16154218

[22] Chung HT , Lee B , Park E , Lu JJ , Xia P . Can all centers plan intensity‐modulated radiotherapy (IMRT) effectively? An external audit of dosimetric comparisons between three‐dimensional conformal radiotherapy and IMRT for adjuvant chemoradiation for gastric cancer. Int J Radiat Oncol Biol Phys. 2008;71(4):1167‐1174. doi:10.1016/j.ijrobp.2007.11.040. Jul 15. 18234440

[23] Giglioli FR , Strigari L , Ragona R , et al. Lung stereotactic ablative body radiotherapy: a large scale multi‐institutional planning comparison for interpreting results of multi‐institutional studies. Phys Med. 2016;32(4):600‐606. doi:10.1016/j.ejmp.2016.03.015. Apr. 27061871

[24] Wei Z , Peng X , Wang Y , et al. Influence of target dose heterogeneity on dose sparing of normal tissue in peripheral lung tumor stereotactic body radiation therapy. Radiat Oncol (London, England). 2021;16(1):167. doi:10.1186/s13014-021-01891-6 PMC8404286 34461954

[25] Purdie TG , Bissonnette JP , Franks K , et al. Cone‐beam computed tomography for on‐line image guidance of lung stereotactic radiotherapy: localization, verification, and intrafraction tumor position. Int J Radiat Oncol Biol Phys. 2007;68(1):243‐252. doi:10.1016/j.ijrobp.2006.12.022. May 1. 17331671

[26] International Commission on Radiation Units and Measurements. ICRU Report 83: Prescribing, Recording and Reporting Photon‐Beam Intensity‐Modulated Radiation Therapy (IMRT) Journal of the ICRU. 2010;10(1).

[27] Paddick I . A simple scoring ratio to index the conformity of radiosurgical treatment plans. J Neurosurg. 2000;93(3):219‐222. doi:10.3171/jns.2000.93.supplement. Technical note. Dec. Suppl. 11143252

[28] Barriger RB , Forquer JA , Brabham JG , et al. A dose‐volume analysis of radiation pneumonitis in non‐small cell lung cancer patients treated with stereotactic body radiation therapy. Int J Radiat Oncol Biol Phys. 2012;82(1):457‐462. doi:10.1016/j.ijrobp.2010.08.056. Jan 1. 21035956

[29] Chang JY , Li QQ , Xu QY , et al. Stereotactic ablative radiation therapy for centrally located early stage or isolated parenchymal recurrences of non‐small cell lung cancer: how to fly in a “no fly zone”. Int J Radiat Oncol Biol Phys. 2014;88(5):1120‐1128. doi:10.1016/j.ijrobp.2014.01.022. Apr 1. 24661665

[30] Lu C , Lei Z , Wu H , Lu H . Evaluating risk factors of radiation pneumonitis after stereotactic body radiation therapy in lung tumor: meta‐analysis of 9 observational studies. PLoS One. 2018;13(12):e0208637. doi:10.1371/journal.pone.0208637 30521600 PMC6283643

[31] Pollom EL , Chin AL , Diehn M , Loo BW , Chang DT . Normal tissue constraints for abdominal and thoracic stereotactic body radiotherapy. Semin Radiat Oncol. 2017;27(3):197‐208. doi:10.1016/j.semradonc.2017.02.001. Jul. 28577827

[32] Madani I , Vanderstraeten B , Bral S , et al. Comparison of 6 MV and 18 MV photons for IMRT treatment of lung cancer. Radiother Oncol. 2007;82(1):63‐69. doi:10.1016/j.radonc.2006.11.016. Jan. 17182143

[33] Ekstrand KE , Barnes WH . Pitfalls in the use of high energy X rays to treat tumors in the lung. Int J Radiat Oncol Biol Phys. 1990;18(1):249‐252. doi:10.1016/0360-3016(90)90290-z. Jan 2105286

[34] Henry J , Moreno C , Crownover RL , Baacke D , Papanikolaou N , Gutierrez AN . Dosimetric quantification of dose fall‐off in liver SBRT planning using dual photon energy IMRT. J Radiosurg SBRT. 2016;4(2):145‐151. 29296439 PMC5658876

[35] Bender ET . Increasing dose gradient and uniformity in small fields using modulation: theory and prototypes for cone‐based stereotactic radiosurgery. Med Phys. 2014;41(5):051706. doi:10.1118/1.4870380. May. 24784372

[36] Zhang Q , Zheng D , Lei Y , et al. A new variable for SRS plan quality evaluation based on normal tissue sparing: the effect of prescription isodose levels. Br J Radiol. 2014;87(1043):20140362. doi:10.1259/bjr.20140362. Nov. 25226047 PMC4207160

[37] Rusthoven KE , Kavanagh BD , Cardenes H , et al. Multi‐institutional phase I/II trial of stereotactic body radiation therapy for liver metastases. J Clin Oncol. 2009;27(10):1572‐1578. doi:10.1200/JCO.2008.19.6329. Apr 1. 19255321

